# A Narrative Review of Speech and EEG Features for Schizophrenia Detection: Progress and Challenges

**DOI:** 10.3390/bioengineering10040493

**Published:** 2023-04-20

**Authors:** Felipe Lage Teixeira, Miguel Rocha e Costa, José Pio Abreu, Manuel Cabral, Salviano Pinto Soares, João Paulo Teixeira

**Affiliations:** 1 Research Centre in Digitalization and Intelligent Robotics (CEDRI), Instituto Politécnico de Bragança, Campus de Santa Apolónia, 5300-253 Bragança, Portugal; 2 Engineering Department, School of Sciences and Technology, University of Trás-os-Montes and Alto Douro (UTAD), Quinta de Prados, 5000-801 Vila Real, Portugal; 3 Faculty of Medicine of the University of Coimbra, 3000-548 Coimbra, Portugal; 4 Hospital da Universidade de Coimbra, 3004-561 Coimbra, Portugal; 5 Institute of Electronics and Informatics Engineering of Aveiro (IEETA), University of Aveiro, 3810-193 Aveiro, Portugal; 6 Intelligent Systems Associate Laboratory (LASI), University of Aveiro, 3810-193 Aveiro, Portugal; 7 Laboratório para a Sustentabilidade e Tecnologia em Regiões de Montanha (SusTEC), Instituto Politécnico de Bragança, Campus de Santa Apolónia, 5300-253 Bragança, Portugal

**Keywords:** schizophrenia, speech, EEG, ERP, features, emotional state

## Abstract

Schizophrenia is a mental illness that affects an estimated 21 million people worldwide. The literature establishes that electroencephalography (EEG) is a well-implemented means of studying and diagnosing mental disorders. However, it is known that speech and language provide unique and essential information about human thought. Semantic and emotional content, semantic coherence, syntactic structure, and complexity can thus be combined in a machine learning process to detect schizophrenia. Several studies show that early identification is crucial to prevent the onset of illness or mitigate possible complications. Therefore, it is necessary to identify disease-specific biomarkers for an early diagnosis support system. This work contributes to improving our knowledge about schizophrenia and the features that can identify this mental illness via speech and EEG. The emotional state is a specific characteristic of schizophrenia that can be identified with speech emotion analysis. The most used features of speech found in the literature review are fundamental frequency (F0), intensity/loudness (I), frequency formants (F1, F2, and F3), Mel-frequency cepstral coefficients (MFCC’s), the duration of pauses and sentences (SD), and the duration of silence between words. Combining at least two feature categories achieved high accuracy in the schizophrenia classification. Prosodic and spectral or temporal features achieved the highest accuracy. The work with higher accuracy used the prosodic and spectral features QEVA, SDVV, and SSDL, which were derived from the F0 and spectrogram. The emotional state can be identified with most of the features previously mentioned (F0, I, F1, F2, F3, MFCCs, and SD), linear prediction cepstral coefficients (LPCC), linear spectral features (LSF), and the pause rate. Using the event-related potentials (ERP), the most promissory features found in the literature are mismatch negativity (MMN), P2, P3, P50, N1, and N2. The EEG features with higher accuracy in schizophrenia classification subjects are the nonlinear features, such as Cx, HFD, and Lya.

## 1. Introduction

Mental health is a dynamic state of internal balance that enables individuals to use their abilities harmoniously with society’s universal values, their basic cognitive knowledge, and their social skills and to express, modulate, and acknowledge their emotions. Mental health can be influenced by adverse life situations, social functions, and the relationship between the body and mind [1,2].

People with severe mental illness have a reduced average life expectancy of 10 to 20 years compared to the general population, especially if the disease is chronic [3]. Schizophrenia is a chronic and severe mental illness with heterogeneous presentations [4] that directly affects the lifestyles of more than 20 million adults worldwide [5,6].

According to the Diagnosis and Statistical Manual of Mental Disorders, fifth edition (DSM-5), schizophrenia disorders can be defined as “abnormalities in one or more of the following five domains: delusions, hallucinations, disorganized thinking (speech), grossly disorganized or abnormal motor behavior (including catatonia), and negative symptoms” [7].

Making an early diagnosis of a mental illness helps to provide proper treatment and recovery for patients. Current methods of diagnosing schizophrenia are based on observations and interviews conducted by psychiatrists. However, this method can be prone to human error and very time-consuming [8].

Evaluating the cognitive system via characteristics such as attention, perception, memory (functional and explicit), cognitive control, and speech behavior allows the characterization of mental disorders [9].

Speech and language contain relevant information about human thought. For components such as semantic and emotional content, semantic coherence (meaning), structure, and syntactic complexity can be analyzed [10]. Additionally, speech is a complex signal of variable duration that carries both linguistic information and emotion [11]. Cognitive and thought disorders manifest in how speech is produced and what is said [12]. Linguistic disorganization may be neurologically underpinned by the dysfunction of the non-dominant hemisphere, such as in epilepsy, which is lateralized in the non-dominant hemisphere [13,14].

Due to a low cost, relatively easy access, and non-invasive means (although schizophrenia subjects may hardly accept them), electroencephalograms (EEG) are commonly used to diagnose schizophrenia. EEGs are electrical signals that describe brain activity with good temporal and spatial resolution [8].

Some features can be similar to other disorders, which can limit technological advances. The knowledge of speech features and EEG biomarkers associated with schizophrenia is essential to help diagnostics.

According to the American Society for Clinical Pharmacology and Therapeutics, a biomarker is defined as “a characteristic that is objectively measured and evaluated as an indicator of normal biological processes, pathogenic processes, or pharmacological responses to a therapeutic intervention”. These biomarkers serve as a tool to affect prognoses [15].

Technological solutions in mental health may consider several ethical and liable issues and respect the patient’s privacy. The authors of [16] suggest that implementing AI in the psychiatric area can be critical because of different opinions between doctors and researchers.

The medical diagnostic support tools cannot be seen as a final diagnostic but can be seen as a support tool, and the diagnosis is still under medical responsibility.

Performing an early diagnosis of a mental illness helps to provide proper treatment and recovery for patients. Current methods of diagnosing schizophrenia are based on observations and interviews conducted by psychiatrists. In the context of schizophrenia, several evaluation scales are used to differentiate the severity of the diagnosis. The most common are the Negative Symptom Assessment (NSA), the Brief Psychiatric Rating Scale (BPRS), the Scale for the Assessment of Negative Symptoms (SANS), and the Positive and Negative Syndrome Scale (PANSS).

The NSA was initially created with 25 items and was later changed by Axelrod et al. in 1993 [17] to 16 items, becoming the most widely used version. These items are based on communication, social engagement, affect/emotion, motivation, and retardation. Each item and global negative symptoms are rated on a 1-to-6-point scale in which “1” represents no reduction in normal behaviors associated with the item and “6” represents a severe reduction in or absence of the behavior. The rating scale also includes a ”9” for a “not ratable” definition [18]. The main limitation of the NSA is its high reliance on functioning or behaviors, for which severity is measured by the type and frequency of social interactions. The NSA’s 16 items include a prolonged time to respond, restricted speech quantity, impoverished speech content, inarticulate speech, emotion: reduced range, affect: reduced modulation, affect: reduced display, reduced social drive, poor rapport with the interviewer, reduced sexual interest, poor grooming and hygiene, a reduced sense of purpose, reduced hobbies and interests, reduced daily activity, reduced expressive gestures, and slow movements.

The BPRS was used to assess clinical symptoms. The BPRS is an 18-item scale. Each item or symptom is rated discretely from “1” (not present) to “7” (extremely severe). Two calibrated raters conducted the BPRS session using a semi-structured clinical interview in [18,19]. The items of the BPRS are somatic concern, anxiety, emotional withdrawal, conceptual disorganization, guilt feelings, tension, mannerisms and posturing, grandiosity, depressive mood, hostility, suspiciousness, hallucinatory behavior, motor retardation, uncooperativeness, unusual thought content, and blunted affect.

The SANS is one of the most widely used symptom rating scales for schizophrenia. The SANS is a clinician-administered scale developed to ascertain and quantify negative symptoms in inpatients and outpatients with schizophrenia. The SANS comprises 25 negative symptoms representing five domains: affective flattening or blunting (unchanging facial expression, decreased spontaneous movements, paucity of expressive gestures, poor eye contact, affective nonresponsivity, inappropriate affect, lack of vocal inflections, and global rating of affective flattening), alogia (poverty of speech, poverty of content of speech, blocking, increased latency of response, and global rating of alogia), avolition/apathy (grooming and hygiene, impersistence at work or school, physical anergia, and global rating of avolition/apathy), anhedonia/asociality (recreational interests and activities, sexual activity, ability to feel intimacy and closeness, relationships with friends and peers, and global rating of anhedonia/asociality), and attention disturbances (social inattentiveness, inattentiveness during mental status testing, and global rating of attention,). Each item is scored on a six-point scale (0–5) with higher scores indicating greater severity of negative symptoms [20]. The main disadvantage is a single focus on the positive and negative symptoms.

The PANSS is among the best-validated instruments to assess positive, negative, and general psychopathology associated with schizophrenia. The PANSS is a standardized clinical interview that assesses the presence and severity of positive and negative symptoms and general psychopathology for people with schizophrenia within the last week. Of the 30 items, seven are positive, seven are negative, and sixteen are general psychopathology symptoms. The PANSS item are divided into three categories: the positive scale, which includes delusions, conceptual disorganization, hallucinatory behavior, excitement, grandiosity, suspiciousness, and hostility; the negative scale, which includes blunted affect, emotional withdrawal, poor rapport, passive–apathetic social withdrawal, difficulty in abstract thinking, lack of spontaneity and flow of conversation, and stereotyped thinking; and the general psychopathology scale, which includes somatic concern, anxiety, guilt feelings, tension, mannerisms and posturing, depression, motor retardation, uncooperativeness, unusual thought content, disorientation, poor attention, lack of judgment and insight, disturbance of violation, poor impulse control, preoccupation, and active social avoidance. The symptom severity for each item is rated according to the anchor points on the 7-point scale (1 = absent; 7 = extreme) [21]. The main disadvantages of PANSS are the lack of sensitivity in predicting global cognitive functioning, the failure to differentiate between negative symptoms and depression, and frequently incorrect calculations.

Although the medical community accepts these scales, they have some limitations, such as subjectivity, in which the opinion may not be the same among those who assess the symptoms, thus hindering an accurate evaluation. The complexity and variation of symptoms may vary from person to person, and it is not possible to define a pattern among individuals diagnosed, thus compromising how to accurately quantify or measure the symptoms, which may affect confidence in the scales. Cultural and linguistic barriers, a lack of standardization between the different scales, the time taken to make an assessment based on the available scales, and their coverage of symptoms (the scales may not assess all the feelings present in the individual) also call into question the performance of the scales. It is convenient to combine these scales with more clinical features to mitigate these disadvantages.

The benefits of using speech and EEG features as a supplement to the scales currently in use include objectivity, as the features are measured with specialized equipment, reducing the influence of subjective factors (such as differences in opinion between clinicians); sensitivity, as the evaluation of speech and EEG features may be more sensitive than a scale based on human auditory perception; and, finally, ease of application, making it a tool to support medical diagnoses.

The development of systems based on AI using biological features such as the ones discussed in this work may contribute in the near future to improve accuracy, allow the diagnosis with less time and resources, and contribute to preventing psychotic breaks.

This manuscript presents the research on the most used speech features and EEG biomarkers described in the literature for the diagnosis of schizophrenia [22].

The manuscript is organized into five sections. The current section contextualizes schizophrenia and its recognition and presents a short overview of the usage of machine learning methodologies in pathology identification/diagnosis. Section 2 presents the methodology used to carry out the narrative literature review. Section 3 contains theoretical information about speech biomarkers, state-of-the-art developed works with speech features, and an approach to speech emotion detection. The methods using EEG in schizophrenia and corresponding biomarkers are discussed in Section 4. This section includes theoretical information about ERP biomarkers in schizophrenia. The most used features are described in Section 3 and Section 4. Finally, the Discussion and Conclusions section highlights how speech in schizophrenia manifests itself, how the speech/EEG files are collected, and the features of speech and EEG that might be promising for identifying schizophrenia in future applications.

### Machine Learning Classification

**Figure 1:**
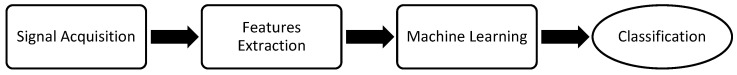
Block diagram for the approach in schizophrenia classification.

Using machine learning, an increasing number of researchers attempt to distinguish patients with schizophrenia from healthy people. Figure 1 shows the generic block diagram for an approach to schizophrenia suggestion or classification.

The feature acquisition step aims to collect data (speech files or EEG biomarkers). It is possible to perform signal preprocessing in this step. The preprocessing methods remove corrupted or noisy signals [23].

The acquisition of speech files is much easier than EEG, but there is one shortcoming: a recording audio contains at least two different voices (patient and interviewer). However, it does not occur when the task is reading. To control this, many tools, such as a Voice Activity Detector (VAD) and diarization methods, are now available to remove unwanted information (for example, the voice of medical staff or background noise).

The EEG is a non-linear and non-stationary signal that requires different signal processing operations to cancel different types of noise artefacts, such as systematic blinking and thermal noises. The Fourier transform could not represent the temporal evolution of this type of signal. Different time–frequency domain-based approaches, such as a short-time Fourier transform or a wavelet transform, are applied to address this issue. A stimulus for the oddball auditory paradigm is typically used for EEG acquisition.

The feature extraction field is an essential process because the information from it can compromise the success of a developed model. It is crucial to extract a clean signal (without artefacts).

Because of the variability of speech signals, feature extraction in speech files is difficult. Generally, signal-processing techniques such as framing, which consists of dividing the original signal into blocks (frames), are used. Thus, in each segment, different types of features are extracted. Some authors apply techniques, such as PSD, to map the EEG files in higher dimensional spaces. On the other hand, the majority of authors use a decomposition method (e.g., discrete wavelet transform) and divide the files into sub-bands to calculate the energy of each cerebral area.

In machine learning, various tools are tested with multiple combinations of settings to find the best solution for the problem of suggestion or classification.

## 2. Methodology

The literature review on speech and EEG features for the diagnosis of schizophrenia was carried out on four databases: Scopus, Web of Science (WoS), PubMed, and IEEE Xplore. We considered published works between 2010 and 2021.

For the speech features, the inclusion criteria had to be mentioned in the title or abstract. Any topic outside this study’s main goal (e.g., the study of medication effect or therapeutic evaluation) was excluded. The inclusion criteria for the speech part were schizophrenia, speech or voice, features or parameters, detection or prediction, and machine learning. A total of 11 references were obtained in Scopus and 10 in WoS. The keywords on the IEEE Xplore and PubMed databases were “schizophrenia and speech features”. The search received 10 references on IEEE Xplore and 6 references on PubMed. Of the 37 results found, 11 were the same paper retrieved from different sources and 2 were discarded because the documents were unavailable. Four more papers known by the authors were included, resulting in a total of 28 articles.

For EEG biomarkers, the inclusion criteria used were “schizophrenia and features” and “EEG” and the search was limited to the title and the abstract. The search found 9 references in Scopus, 8 in the WoS, 10 in PubMed, and 3 in the IEEE Xplore. Of the 30 results, 2 from Scopus and 2 from WoS were discarded because of unavailability, 5 because they did not fit into the research context, and 3 because they were repeated in databases. Therefore, only 18 articles remained.

## 3. Speech Features

Speech contains several measurable features that indicate aspects of cognitive health [12], so acoustic changes observed in subjects with schizophrenia are conceptualized as a component of negative symptoms [24,25,26].

Prosodic features permit the identification of the type of component of speech that refers to the way words are spoken and also show the characteristics of the speaker or speech. In other words, they show whether a statement is an affirmation or a question, whether irony or sarcasm is present, in addition to contrast, emphasis, and focus [24].

Language can be influenced by four factors: the participants, the situation, the conversation topic, and the speaker’s function [27]. For identifying schizophrenia via speech in two different languages, it is essential to understand that some languages have faster speakers than others (for example, Japanese or Spanish speakers speak faster than Mandarin speakers).

In schizophrenia, negative symptoms are divided into five domains: blunted affect, asociality, alogia, anhedonia, and avolition [28]. Blunted affect and alogia are defined in terms of reduced vocal prosody [29], asociality consists of reduced social activities and interactions, anhedonia is diminished experience of pleasure related to current or future activity, and avolition is diminished motivation and lack of drive resulting in reduced initiation and maintenance of goal-directed activities [30].

Speech in subjects with schizophrenia is perceived as a negative symptom because it is mainly reflected in a lack of emotion (blunted affect) and poor speech (alogia) [24].

Other speech symptoms in schizophrenia include slow speech, reduced pitch variability, more pauses, and less synchronization in syllable variability [25,28].

Concerning features related to semantic analysis, schizophrenic speech was less semantically coherent and less connected [31]. Incoherence is also typical in schizophrenia, although there may be tangentiality (irrelevant answers to questions), derailment (loss of association between two sentences), illogical speech, and indirect speech [32].

An emotional state can be an important biomarker in schizophrenia [28], and it is possible to recognize emotions with automatic analysis via speech [33]. Emotions consist of psychological states that derive from different strands, such as personal experiences, physiological conditions, and behavioral and communicative reactions [11].

The last paragraphs identified three levels of features expressed in speech. Features extracted directly from the speech level include prosodic, spectral, temporal and statistic features. Features extracted from the semantic features level include coherence, connected speech, derailment, illogical speech, and indirect speech. Finally, the other features are related to the level of emotional state.

Speech production in patients with schizophrenia is usually stimulated via clinical interviews [27,32], free speech activities [26,33,34,35,36], image description [37,38,39] or reading [32,34,40]. Free speech can be compromised in people with a diagnosis of schizophrenia. Therefore, techniques such as asking patients to report activities or plans for the future [41,42], tasks done the previous day [43], and dreams [44] can be implemented. Narrative of Emotions Tasks can also be used during medical consultation [32].

It is possible to obtain speech using mobile phones, computers, and tablets, which is more advantageous compared to neuroimaging methods that can be more expensive and invasive [2].

The features in the temporal domain are simple to relate to the perceptive properties of speech, thus making it a better infrastructure for further use by the psychiatric community. Some works have used these features, including [43,44].

Acoustic feature extraction can be done with software/algorithms such as OpenSmile, Covarep, pyAudioAnalysis, OpenEAR, and Praat [2].

Although all the studied works use continuous speech, some authors have used parameters (jitter and shimmer) that are more suited to be extracted from sustained speech. As such, the recordings can be divided into clips with short durations [45].

Using speech as a biomarker has the following advantages: it is difficult to hide symptoms; it directly expresses emotion and thought via its linguistic content; it indirectly reflects neural modulation via motor and acoustic variation; and its set of advantages can be generalized to different languages because vocal anatomy is similar.

### 3.1. State of the Art (Speech)

Considering what has been reviewed thus far, the works that use speech to diagnose schizophrenia are limited, which may be due to the difficulty in obtaining authorization to collect a speech dataset. Typically, the authors record the dataset used. However, within this limitation, most of the works, as in the case of [28,46], are based on speech in a natural context. Speech patterns in schizophrenia have also been analyzed in some works but to a lesser extent, as in the case of [46].

According to this study, the features used to identify schizophrenia are divided into four categories: prosodic, spectral, temporal, and statistical features. However, in addition to these characteristics, a quantitative measure, namely, the number of words (verbosity), was used.

Gosztolya et al. [43] used only temporal features obtained from spontaneous speech, such as articulation rate, speech tempo, duration of utterance, number of pauses, duration of pauses, pause duration rate, pause frequency, and average pause duration. The authors achieved 70–80% accuracy in classifying subjects with schizophrenia diagnosis [43].

Other authors used two categories of features. Kliper et al. [24] used temporal and prosodic features to identify schizophrenia, depression and control. The parameters used include spoken ratio, utterance duration, gap duration, pitch range, the standard deviation of pitch, power standard deviation, mean waveform correlation, mean jitter, and mean shimmer. These parameters allowed the classification of control vs. schizophrenia with an accuracy of 76.19%, control vs. depression with an accuracy of 87.5%, and schizophrenia vs. depression with an accuracy of 71.43%. For multiclass classification they achieved 69.77%.

Martínez-Sánchez et al. [42] and Rapcan et al. [47] showed that patients with schizophrenia tend to have slow speech, reduced pitch variability, and a more significant number of pauses. Rapcan et al. [47] investigated the fundamental frequency (F0) and the relative variation of vocal pitch and, using temporal and prosodic features, attempted to study the total speech duration but did not find a statistical significance and argued that the lack of academic qualifications of the subjects under analysis compromised the results.

Compton et al. [26] also used two categories of features but, in their case, used prosody and spectral categories. They studied schizophrenic patients and healthy subjects with and without aprosody. They concluded that patients with aprosody present lower F0, F2, and intensity/loudness values.

The severity of negative symptoms in the first outbreak of schizophrenia is correlated with the second-order formant F2. This conclusion was obtained after the study with fundamental frequency F0 and the first and second-order formants F1 and F2 [34].

He et al. [48] also detected negative symptoms using the parameters: symmetric spectral difference level (SSDL), quantification error and vector angle (QEVA), and standard dynamic volume value (SDVV), thus discriminating subjects with and without a diagnosis of schizophrenia with an accuracy of 98.2% (with decision trees).

Other authors used three categories of speech features. To identify cognitive and thought disorders. Voleti et al. [12] tried to find acoustic features of speech. These disorders include various neurological impairments (e.g., dementia) and psychiatric conditions (e.g., schizophrenia). Prosodic articulation temporal and vocal quality features were used. Temporal features include the duration of voiced segments and the duration of silent segments. The prosodic features covered loudness, periodicity measures, and F0. The spectral or articulation features comprise formant frequencies (F1, F2, and F3) and MFCCs. They also used jitter, shimmer, and harmonic-to-noise ratio (HNR) features.

Parola et al. [49] analyzed three categories of parameters, qualitative indices, quantitative analysis, and multivariate machine learning (ML) tools. Using ML, the results are more promising. For schizophrenia and healthy identification, free speech-based studies provide higher differences between groups.

Some authors used features of all four categories. Agurto et al. [45] could predict psychosis with 90% accuracy using prosodic, spectral, temporal, and statistical measures. The feature set was formed for spectral characterization by MFCCs, spectral slope, interquartile range (IQR), maximum energy, and frequency. For vowel characterization, they used F1, F2, and F3 (frequencies and their corresponding bandwidth). For voice quality, they used jitter (local absolute value and ppq5), shimmer (local absolute and apq5), autocorrelation, harmonic-to-noise ratio (HNR), and noise-to-harmonic ratio (NHR). For the rhythm changes, pauses (threshold of −25 dB and minimum duration of 100 ms) and voiced parts were considered. For each category mentioned above, the authors calculated the median, IQR, pct5, pct95, skewness, kurtosis, total interventions, speech rate, articulation rate, and speech/non-speech ratio (and corresponding percentages). In addition, they calculated speech rate/velocity of speech and articulation rate. These equations are indicators of cerebral activity, and, using them, it is possible to obtain a cerebral processing rate.

Tahir et al. [4] state that using a Multi-Layer Perceptron (MLP) neural network classifier allows an assessment of negative symptoms using the features of speaking rate, frequency, and volume entropy. The author also experimented with other types of features such as prosodic features (F0), spectral features including first, second, and third order formants (F1, F2, F3), MFCCs, amplitude (minimum, maximum, and mean volume), conversational/temporal features including duration of speech, speaking turns, interruptions, interjections, and statistical features such as entropy.

Similar to the aim of the previous study, Low et al. [2] concluded that features such as peak slope, linear predictive coefficients, and mean pause duration are directly correlated with schizophrenia. Quasi-open Quotient—QOQ, F1 range, articulation rate, pause rate, speech rate, time talking, and mean intensity are negatively correlated with schizophrenia. Moreover, the parameters including total number of pauses, mean speech duration, intensity variability, and F0 variability, among others, despite being used in many studies, do not show any correlation with schizophrenia.

Other authors used the semantic level of features. Mota et al. [31] evaluated the structural characteristics of each interview conducted so that each interview was converted into a graph in which each word is represented by a node and the temporal sequence between two words is represented by an edge (margin). The same procedure was performed every 30 consecutive words to analyze the verbosity. After this procedure, the authors evaluated the number of edges (margins) and the node connection. For semantic analysis, the median semantic distance between two sentences was calculated using latent semantic analysis (LSA). The authors stated that schizophrenia speech produces fewer linked words and less semantic coherence via the structural and semantic features.

On the other hand, for the prediction of psychotic outbreaks (in young people at high clinical risk—CHR), Bedi et al. [50] evaluated semantic and syntactic features. They detected two features in semantic coherence: the minimum semantic distance for first-order coherence (e.g., the minimum coherence or maximum discontinuity between two sentences) and the average semantic distance for first-order coherence (e.g., the average coherence between sentences). With the studied features, the authors could predict the development of psychosis with 100% accuracy.

The formal linguistic aspects of auditory verbal hallucinations (AVHs) indicate that speaking in the first person is less common in hallucinated speech. Sentences have no grammatical connectivity; speech has no connection and, usually, it is personalized. Thus, although there are individual variations, there is a linguistic profile of typical speech in people with verbal auditory hallucinations [51].

Some works combine speech acoustic features with text features. Xu et al. [52] transcribed the interviews (with software help), so it was possible to use speech and text parameters. The verbal speech parameters were LIWC, diction, Latent Dirichlet Allocation, and Doc2vec features. The non-verbal speech parameters were composed of conversational, OpenSmile, and DisVoice elements, thus distinguishing diagnosed and undiagnosed subjects with an accuracy of 76.2% [52,53].

One work’s authors [47] suggest that the lack of academic qualifications can compromise studies in this context. To increase performance, techniques could be applied as suggested in [17], in which speech is transcribed and text parameters are used simultaneously with the speech parameters.

Speech analysis was also combined with other parameters. In [28], the algorithm’s performance increased when body movements were implemented as input parameters. For example, [28] applied low-level descriptors (LLD) and body movements to detect negative symptoms. The LLD set is composed of intensity, loudness, MFCC (12), pitch (F0), probability of voicing, F0 envelope, 8 LSF (Line Spectral Frequencies), and Zero-Crossing Rate. Using an SVM classifier with the LLD alone, the authors obtained an accuracy of 79.49%. If these features were combined with body movements, the accuracy improved to 86.36%.

Feature selection procedures were also implemented. To make a selection of the most promising parameters in the identification of schizophrenia via speech, Espinola et al. [25] used the Particle Swarm Optimization (PSO) method. Out of a set of 33 features, zero-crossing rate, Hjorth parameter complexity, average amplitude changes, mean absolute value, kurtosis, third and fourth moments, maximum amplitude, peak frequency, power spectrum ratio, mean, and total power (12 out of 33) were selected. With SVM, the authors reached an accuracy of 91.79% in classifying subjects with and without a diagnosis of schizophrenia.

Argolo et al. [53] concluded that structured interviews or task descriptions are the most commonly used for automated speech evaluation in these studies, similarly to studies based on free speech.

One of the most used machine learning tools is SVM, which has an accuracy rate between 70% and 91.79%. Using MLP, one author [4] obtained an accuracy of 81.3%. Utilizing Linear Discriminant Analysis (LDA), the authors of [47] achieved 79.4% accuracy. Using Signal Processing Algorithms, the authors of [42] achieved 93.8% accuracy in the discrimination between patients and controls. With decision trees, another author [48] obtained 98.2%. Lastly, the best accuracy achieved was obtained in the work of [50], which had an accuracy of approximately 100%, but for the prediction of psychotic outbreaks.

Although the set of previously analyzed features can indicate typical characteristics of schizophrenia, they do not identify schizophrenia exclusively. Other mental disorders or an anatomic deformation in the vocal tract can compromise these features. Therefore, the combination of several features is required for a schizophrenia diagnosis.

A summary of the features most used in the literature is presented in Table 1. The most frequently used speech parameters are divided into four main categories. The prosodic category features mostly used are F0, Intensity/Loudness/Amplitude, Jitter, and Shimmer. In the spectral category, the features more frequently used are frequency formants F1, F2, and F3 and MFCCs. The temporal features mostly used are utterance duration, the duration of pauses, and the number of pauses. For quantitative measures, some authors, such as [31,45,54], suggest that the number of pauses can be promising. Finally, the statistical features mostly used are the number of words and verbosity.

Table 2 shows the parameters used by several authors organized according to the categories to which they belong. Not all of the authors mentioned in Table 2 attempted to identify schizophrenia via speech; therefore, the “accuracy” column contains a short description. In the case of these authors, no accuracy was reported, and the table presents only theoretical conclusions. In work [50], the authors achieved 100% accuracy but in classifying psychotic outbreaks in young people at CHR. Therefore, this work is excluded from this accuracy comparison.

Using a single category, the accuracy varies between 80 and 93% (the temporal and statistical features, respectively) No author has used the category of spectral parameters alone.

The most common approach is to use a combined set of parameters (two or more categories). With two categories, the best result obtained was using prosodic and spectral parameters, as in the work of [48] (98% accuracy). Using three categories, the best result was obtained with prosodic, spectral, and temporal features (92% accuracy in [25]). Using the four categories, the maximum accuracy of 90% was achieved in two works.

The use of temporal features alone does not present a discriminant power that can be considered for the identification of schizophrenia, and similarly to other authors, it will be an advantage to combine at least two categories of parameters. The more promising category are the prosodic and spectral features.

The prosodic features F0 and its derived ones, such as QEVA, SDVV, and the spectral SSDL (derived from the spectrogram), have the best performance in schizophrenia classification.

### 3.2. Speech Features Description

This section describes the speech features mentioned previously.

The fundamental frequency (or pitch) measures the frequency of vibration of the vocal folds; consequently, its inverse is the fundamental or glottal period. There are several methods for estimating the fundamental frequency. The most robust is estimating the first peak of the normalized autocorrelation of the signal [59].

The intensity (loudness or amplitude) is defined as the acoustic intensity in decibels relative to a reference value and is perceived as loudness [2].

Jitter measures deviations in frequency between consecutive glottal periods, and this commonly used method is based on the DSYPA algorithm (dynamic programming project phase slope algorithm). This algorithm estimates the opening and closing instants of the glottis (glottal closure instant) [59]. Jitter can be measured in four different ways, but the most used ways are relative jitter (jitter) and absolute jitter (jitta). Relative jitter is the mean absolute difference between the consecutive glottal periods divided by the mean period and is expressed as a percentage. The absolute jitter is the variation of the glottal period between cycles (the mean absolute difference between consecutive periods) [60].

The shimmer is related to the magnitude variation along the glottal periods, which can be measured in four different ways. Relative Shimmer (Shim) and Absolute Shimmer (ShdB) are the most used. Relative Shimmer is defined as the mean absolute difference between the magnitudes of consecutive periods divided by the mean magnitude and is expressed as a percentage. The Absolute Shimmer is expressed as the peak-to-peak magnitude variation in decibels [60].

The remaining determinations forms of jitter and shimmer are not used because in a statistical study carried out by [61] they did not show statistically significant differences between jitter and relative shimmer correspondingly.

The Harmonic-to-Noise Ratio (HNR) measures the ratio between harmonic and noise components, quantifying the relationship between the periodic component (harmonic part) and aperiodic components (noise). HNR can be measured by the ratio between the amplitude of the first peak of the normalized autocorrelation, considering that this is the energy of the harmonic component of the signal, and its difference to one, that is the noise energy. This feature can be obtained with Equation (1), where H is the harmonic component given by the energy of the signal’s first peak of the normalized autocorrelation. The final value of HNR is the average along all segments [60].

(1) $$\mathrm{HNR}\left(\mathrm{dB}\right)=10*\log_{10}\frac{H}{1-H}$$

The Noise-to-Harmonic Ratio NHR can be calculated by Equation (2). To determine the autocorrelation, it is necessary to multiply the normalized autocorrelation of a segment of a speech signal by the normalized autocorrelation of a window (ex. Hanning window). Then, the first peak of the segment signal is the autocorrelation.

(2) NHR=1−Autocorrelation

The Quantization Error and Vector Angle (QEVA) contain two indicators, the mean value of the cumulative error and the mean value of the vector angle. Both indicators are calculated based on the fundamental frequency curve and fit the fundamental frequency curve. The QEVA permit evaluates the stability and similarity of the successive fundamental frequencies of the speech signals [48].

The Standard Dynamic Volume Value (SDVV) considers the monotonous speed and intensity of speech. Considering the speaking behavior of schizophrenic people, it is related to flat affect in schizophrenic patients. The calculation is divided into three steps. The first step is the intensity calculation based on voice segments (Equation (3)), where Mws represents the intensity of speech, M is the number of voice segments, ω denotes the voice segment, L is the length of one voice segment, i denotes the index of speech content from a speaker, j represents the index of voice segments in the speech content, and r is adopted to regularize the amplitudes of voice segments.

(3) $$M_{\mathrm{ws}}={\left(\frac{1}{\mathrm{ML}}\overset{M}{\underset{i=1}{\sum }}\overset{L}{\underset{j=1}{\sum }}\omega \left(i,j\right)\right)}^{r}$$

The next step consists of determining the normalized exponent variance calculation using Equation (4), where Vs represents the exponent variance in a sentence; s(n) denotes the normalized sentence; $\bar{s\left(n\right)}$ is the mean value of all the data points in the sentence, including those in the word intervals; S_l_ is the length of the whole sentence; and t is also adopted as r.

(4) $$V_{s}={\left(\frac{\sum \left(s(n\right)-{\bar{s\left(n\right))}}^{2}}{S_{l}}\right)}^{t}$$

The last step consists of the standard dynamic volume value calculation using Equation (5). It aims to represent the intensity variations in speech signals more objectively.

(5) $$S\mathrm{DVV}=\frac{S_{l}^{t}}{{\left(\mathrm{ML}\right)}^{r}}\frac{\sum_{i=1}^{M}\sum_{j=1}^{L}w{\left(i,j\right)}^{r}}{\sum {\left(s\left(n\right)-\bar{s\left(n\right)}\right)}^{2t}}$$

The Velocity of Speech and Articulation Rate (Equations (6) and (7)) correspond to the ratio between the number of syllables and the total time recorded with and without the duration of pauses.

(6) $$\mathrm{Velocity}\;\mathrm{of}\;\mathrm{Speech}=\frac{\mathrm{Number}\;\mathrm{of}\;\mathrm{Syllables}}{\mathrm{Total}\;\mathrm{Time}\;\mathrm{Recording}},$$

(7) $$\mathrm{Articulation}\;\mathrm{Rate}=\frac{\mathrm{Number}\;\mathrm{of}\;\mathrm{Syllables}}{\mathrm{Total}\;\mathrm{Time}\;\mathrm{Recording}\;\left(\mathrm{after}\;\mathrm{pause}\;\mathrm{remove}\right)},$$

The peak slope corresponds to the slope of the regression line that fits lof10 of the maxima of each frame [2].

The Mel Frequency Cepstral Coefficients (MFCC) are used to obtain an approximation of the perception of the human auditory system to the frequencies of sound. They are calculated via the frequency spectrum of small windows of the speech signal, which is obtained by the Fast Fourier Transform (FFT). Subsequently, the frequency spectrum is subjected to a bank of triangular filters, equally spaced in the Mel frequency scale, via the discrete cosine transform applied to the output of the filters. Between 13 and 20 coefficients are usually determined. Finally, energy and delta (variations along the sequence of MFCCs speech segments) are calculated [60].

The frequency formants F1, F2, and F3 correspond to the first, second, and third peaks in the spectrum resulting from a human vocal tract resonance.

The linear predictive coding (LPC) coefficients are the best method to predict the values of the next time point of the audio signal using the values from the previous *n* time points, which is used to reconstruct filter properties [2].

Symmetric Spectral Difference Level (SSDL) reflects the distribution of frequency components in the speech spectrum. It is calculated using Equation (8) [48], where N is the number of words in one emotional text; n is the word index; m denotes a factor for adjusting the symmetric amplitude difference; and a is the exponential factor, which constrains the distribution range of SSDL values.

(8) $$\mathrm{SSDL}=\frac{1}{N.10^{a}}\sum_{n=1}^{N}\frac{\sum_{i=1}^{\frac{\mathrm{fs}}{d}-1}{\left|\mathrm{Sn}\left(f\left(\frac{\mathrm{fs}}{d}-i\right)\right)-\mathrm{Sn}\left(f\left(\frac{\mathrm{fs}}{d}+i\right)\right)\right|}^{m}.\mathrm{fn}\left(\frac{\mathrm{fs}}{d}-i\right)}{\mathrm{Cn}},$$

Cn is the inverse of En (Equation (9)):

(9) $$\mathrm{Cn}=\frac{1}{\mathrm{En}}\;\;\;\;\;\;\;\;\;\;\;\;\;\;\;\;\mathrm{En}=\int_{0}^{\mathrm{fs}/d}\mathrm{Sn}\left(\mathrm{fn}\right)\mathrm{dfn}$$

The Zero-Crossing rate (ZCR) is the rate at which the signal changes from positive to negative and back, which is defined in Equation (10), and sgn x(k) in Equation (11).

(10) $$\mathrm{ZCR}=\frac{1}{2N}\overset{N}{\underset{k=1}{\sum }}\left|\mathrm{sgn}\;x\left(k\right)-\mathrm{sgn}\left[x\left(k-1\right)\right]\right|$$

(11) $$\mathrm{sgn}\left[x\left(k\right)\right]=\left\{\begin{array}{c} 1,\;x\left(k\right)\geq 0\\ -1,x\left(k\right)<0 \end{array}\right.$$

The utterance duration corresponds to the time taken to say the utterance, and the number of pauses corresponds to the number of silences in the speech without counting the silence of occlusions in the stop consonants. The duration of pauses corresponds to the time duration of these silences. The gap duration is any segment of recording with no subjects’ speech [24]. The proportion of silence (in percentage) is the relationship between the duration time of all silence segments (without the occlusion of stop consonant) and the total duration of the speech. The total recording time is the total duration of the conversation.

Voiced and unvoiced percentages correspond to the relationship between speech and silence in total time recorded in the discourse. Quasi-open Quotient (QoQ) is the ratio of the vocal folds’ opening time [2]. The number of words and verbosity correspond to the number of words in the discourse. Speaking turns correspond to the number of changes between the speakers in the discourse. The interruption is when someone speaks and is interrupted. The interjection corresponds to a sound that contains no information (e.g., “hmmm”).

The probability of voicing is the probability that speech is present and generally returns a row vector with the same length of speech signal. This value can be obtained with a function such as “voiceActivityDetector” in Matlab Software.

The Interquartile range (IQR) is the difference between the upper and lower quartile in an order data set. The skewness is a measure of the lack of symmetry; the data are symmetrical if it looks the same to the left and right of the center point. The kurtosis is a measure of the relative peakedness of a distribution. The slope sign changes are a statistical feature defined as the number of times the slope of the signal waveform changes sign within an analysis window. The Hjorth feature is divided in three parameters: activity, mobility, and complexity. The activity gives a measure of the squared standard deviation of the amplitude of the signal x(t) (Equation (12)), the mobility represents the mean frequency or the proportion of the standard deviation of the power spectrum (Equation (13)), and the complexity indicates how the shape of a signal is like a pure sine wave and gives an estimation of the bandwidth of the signal (Equation (14)) [62].

(12) $$\mathrm{activity}=\mathrm{var}\left(x\left(t\right)\right)$$

(13) $$\mathrm{mobility}=\sqrt{\frac{\mathrm{activity}\left(x^{\prime }\left(t\right)\right)}{\mathrm{activity}\;\left(x\left(t\right)\right)}}$$

(14) $$\mathrm{complexity}=\frac{\mathrm{mobility}\;\left(x^{\prime }\left(t\right)\right)}{\mathrm{mobility}\left(x\left(t\right)\right)}$$

The minimum and mean semantic distance for first-order coherence are measured as an index of “disorder” in the text [50].

### 3.3. Emotion Detection in Speech

It is not easy to understand human emotions quantitatively, but understanding them is fundamental to human social interactions. The best way to analyze them is by assessing facial expressions or speech [63].

The emotional state is vital for ensuring a good lifestyle and can be influenced by social relations, physical conditions, or health status. Various sources of information such as facial expression, brain signals (EEG), and speech can be used to identify a person’s emotion [63].

There are six basic emotions, including anger, happiness/joy, disgust, surprise, fear, and sadness, and a neutral emotional state. The other emotions are derived from these [54].

Since anhedonia (the inability to feel pleasure or satisfaction), hallucinations, and delirium are symptoms of schizophrenia, the last two of which can be accompanied by strong emotions, these symptoms can lead to a decrease in motivation and a limitation of social life. Hallucinations and delusions can also lead to an increase in anxiety and stress levels.

Emotions are convoluted psychological states composed of several components, such as personal experience and physiological, behavioral, and communicative reactions [11]. Studies with schizophrenic people show that they suffer difficulties in emotional recognition [64].

**Figure 2:**
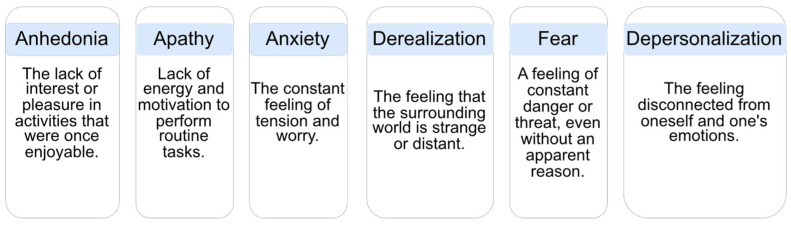
Emotions in schizophrenia.

An emotional state is a feature in patients with schizophrenia [2]. Figure 2 represents the most common emotions in schizophrenia. If possible, finding an emotional state based on speech features may be a further advantage for applications in the future context of this work.

Modulations in pitch [41] often control the emotional state. Most of the relevant developed work is based on using prosodic analysis to recognize emotional features.

Emotion classification is one of the most challenging tasks in speech signal processing [65]. In the work developed in [58], the authors show that acoustic and prosodic information can be combined and integrated with a speech recognition system using suprasegmental states. The same authors state that prosodic information is essential for the reliable detection of a speaker’s emotional state.

Speech emotion recognition (SER) parameters can be divided into acoustic and non-acoustic. Within acoustic, they can be grouped into different categories: prosody, spectral, wavelet, nonlinear, speech quality, and deep learning-based (encoder). The prosody features, mainly derived from F0, discriminate well between high and low arousal emotions (sad and happy). Spectral features extract the energy content of different frequency bands; the most used in emotion recognition are MFCC, Linear Predictive Cepstral Coefficients (LPCC), and Perceptual Linear Prediction (PLP) coefficients. The wavelet-based features provide better temporal resolution for the high-frequency components and better frequency resolution for the low-frequency components. Voice quality features measure the attributes related to the vocal cords (e.g., jitter, shimmer, instantaneous pitch, phase, energy, autocorrelation, harmonic-to-noise ratio (HNR), normalized noise energy (NNE), and glottal noise excitation (GNE)). Nonlinear features capture the complexity of speech signals on different emotions. The most popular are correlation dimension (CD), largest Lyapunov exponent (LLE), Hurst exponent (HE), and Lempel–Ziv complexity. The deep-learning-based features are directly given to a machine learning tool, such as a convolutional neural network (CNN) or a long–short-term memory network (LSTM). The encoder layer of the deep-learning architecture model contains the abstract features of input speech. Non-linguistic features include non-verbal activities, such as laughter or crying, that can be detected using an automatic speech recognition system [66].

Paralinguistic features include attitudinal, intentional, and stylistic information [67]. They are essential for understanding and interpreting the pronunciation and identification of an emotional state [42]. Word choice likely indicates a speaker’s emotional state [58].

For the detection of an emotional state, the MFCCs [68,69,70], zero crossing rate, energy, the entropy of energy, spectral centroid, spectral spread, spectral entropy, spectral flux, spectral roll-off, chroma vector, and chroma deviation [71] were used in previous studies.

Yadav et al. [72] presented a method to detect moments in the emotional state using Zero Time Windowing (ZTW) based on spectral energy. This method sums up the three spectral peaks at each instant of the sample Hilbert envelope of Numerator Group Delay (HNGD).

## 4. EEG in Schizophrenia

Electroencephalography (EEG) provides a non-invasive tool [73] for the study of the brain’s temporal and spatial register of electric activity. The study of schizophrenia via electrophysiological activity focuses on many aspects, including not only finding and explaining deficits but also correlating them with symptoms, cognitive domains, heredity, and even medication [74].

Schizophrenia is a complex and heterogeneous disease, manifesting deficits that underlie many overlapping pathological mechanisms distributed across multiple brain regions. Patients with schizophrenia have sensory processing deficits [75,76,77] and high-level attention-dependent cognitive deficits [78]. These deficits can be assessed by the time-locked EEG activity in stimuli called ERPs [79] and extracting the features. In some studies, such as [80,81,82], EEG signals are recorded with eyes closed and resting using multiple channels, usually at a sampling frequency over 250 Hz [81,82,83].

EEG oscillations are considered biomarkers or features of complex states in health and schizophrenia persons [84]. The oscillatory activity of the EEG in schizophrenia patients indicates abnormal temporal integration and interregional connectivity of brain networks during neurocognitive function [84]. EEG signal analysis can be performed in the time, frequency, and time–frequency domains [85].

Abnormalities in functional and structural networks are associated with schizophrenia, in which they show less dispersion of excitation flow in prefrontal and premotor areas [73] with the prefrontal cortex (a region critical for working memory performance) being a brain region responsible for schizophrenia [83,86].

Brain connectivity can be briefly divided into structural connectivity (SC), functional connectivity (FC), and effective connectivity (EC) [87]. Neural oscillations can be measured by extracranial EEG and represented in specific frequency bands: delta (1–3 Hz), theta (4–7 Hz), alpha (8–12 Hz), beta (13–30 Hz), and gamma (31–80 Hz). EEG signals have shown that there is a significant difference in activity in the theta frequency band and, in particular, an increase in the lower frequencies (delta and theta) in patients with psychotic disorders [79,88]. Delta and alpha frequencies may provide a useful neurophysiological biomarker for delineating psychotic disorders [89]. In patients with schizophrenia, oscillatory disturbances in the gamma wave are also common.

EEG microstate features are appropriate for schizophrenia classification. Microstates segment the EEG signal until a quasi-steady state analysis is obtained [84].

Many studies use machine learning algorithms for computer-aided diagnosis from fMRI and EEG, and in particular, EEG based on functional connectivity [87]. These neuroimaging scans can capture irregularities in functional connectivity in subjects diagnosed with schizophrenia and reflect electrophysiological dysfunction [80].

The following sections present the review of the features extracted from EEG and event-related potential (ERP) EEG biomarkers (understood as a measurable indicator of some biological state or condition) in patients with schizophrenia.

### 4.1. EEG Features

**Figure 3:**
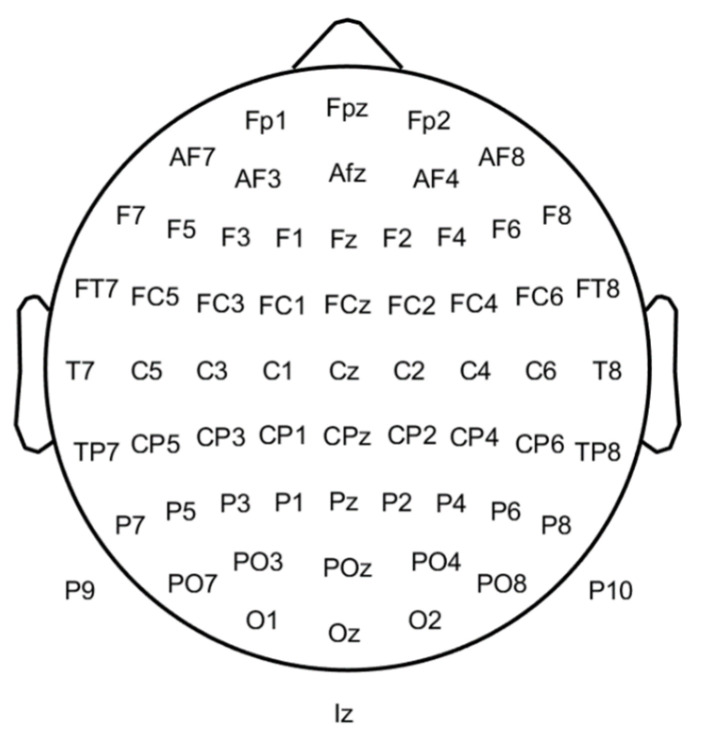
Depiction of the sixty-four electrodes’ layouts on a 2D representation of the scalp.

This section describes the main features that can be used to diagnose schizophrenia with an EEG signal. The spatial position in 64 EEG electrodes can be observed in Figure 3. Additional details about the EEG approach can be found in [90].

To explain the underlying abnormalities in patients diagnosed with schizophrenia, a multi-set canonical correlation analysis (MCCA) was performed by [85] to combine functional magnetic resonance imaging (fMRI), EEG, and structural magnetic resonance imaging (sMRI) parameters. In the work of Shim et al. [80], three sets of parameters were used: sensor-level parameters (124 parameters), source-level parameters (314), and a combination of both.

On the other hand, some authors researched something more specific. Bougou et al. [87] focused on the delta and theta bands (0.5–8.5 Hz) by applying a Butterworth, order 5, band-pass filter to study the connectivity. The authors calculated connectivity measures: cross-correlation (COR), quadratic magnitude coherence (COH), imaginary part of quadratic magnitude coherence (iCOH), phase-locked value (PLV), phase-locked index (PLI), p-index (RHO), transfer entropy (TE), mutual information (MI), Granger causality (GC), partial directed coherence (PDC) and directed transfer function (DTF).

Vittala et al. [73] used transcranial magnetic stimulation (TMS) combined with EEG to alter and measure the neurophysiological parameters of cortical function, including oscillatory activity, cortical inhibition, connectivity, and synchronization.

Using the nonlinear features, including complexity (Cx), Higuchi fractal dimension (HFD), and Lyapunov exponents (Lya), the authors of [91] increased the prediction of a schizophrenia classifier up to 100%. With the decomposition of the EEG into wavelets of six levels (thus creating seven sub-bands), it is also possible to diagnose subjects with schizophrenia with a high accuracy [82].

Based on the phase space dynamics (PSD) of EEG signals C, it can be confirmed that the PSD shape of the Cz channel (Figure 3) in schizophrenia is more regular than in healthy people and can be applied as a biomarker. Via graphical analysis, it is also possible to identify schizophrenia. The PSD maps of signals from the EEG to a higher dimensional space, and the features to be used are extracted with (up to) 19 channels. Generally, the PSD of EEG signals is a suitable technique for discriminating between healthy and schizophrenic groups. Furthermore, the Cz channel is better than other channels at detecting schizophrenia using the PSD of EEG signals [88].

According to Akbari et al. [88], the best accuracy (94.8%) is obtained with graphical features, namely, the summation of distances between Heron’s circular (SDHC), the summation of the shortest distance from each point relative to the 45-degree line (SH45), and the summation of the area of the triangles making successive points and the coordinate center (TACR), as obtained from 12 channels. This procedure was performed by using the phase space dynamic (PSD) of EEG signals. First, the PSD of two EEG signals was plotted on Cartesian space, and then graphical features were extracted to evaluate the chaotic behavior of PSD based on healthy and schizophrenic subjects. The PSD of EEG signals can be transferred to successive triangles. By averaging the coordinates of the corners of each triangle, the centroid coordinate of the triangle is obtained, and it is the same as the centroid of the corresponding Heron’s circle. The SDHC quantifies the variability of the PSD. It can be used to evaluate the complexity of PSD. The SH45 measures the width of the PSD shape from the bisector of the first and third trigonometric regions, and TACR measures the variation rate of the PSD shape of EEG signals [88].

Baygin et al. [8] proposed a model for the automatic detection of schizophrenia based on Collatz conjectures (Collatz conjecture is a mathematical model used in information security applications) using EEG. This model can generate features, is highly accurate, and requires little time to run, allowing it to achieve a 99.47% correct classification. This model comprises three stages. The first consists of a new feature generation with Collatz Conjecture, named the Collatz pattern. Combining the Collatz pattern and the maximum absolute pooling decomposer creates new multilevel features (low-level and high-level features). The second step involves applying the iterative neighborhood components analysis to select the clinically significant features. The last step consists of choosing features fed to the K-nearest neighbors (KNN) classifier for the automated detection of schizophrenia [8].

The hit rates for identifying schizophrenia conditions using EEG parameters range from 82.36% [87] to 100% [91]. Using EEG parameters, the authors of [8] applied a combination of techniques and KNN classifiers, achieving the classification accuracy of 99.47% and 93.58% in two datasets using 19 and 10 channels, respectively. Using various Machine Learning tools such as Support Vector Machine and the leave-one-out cross-validation training procedure, the authors of [91] correctly classified 88.24% of the cases. Random Forest classifier with Direct Transfer Function obtained a correct classification of 82.36% in the work of [87]. The authors of [88] used KNN and a generalized regression neural network (GRNN) and achieved 94.8% accuracy. The maximum accuracy was obtained with a probabilistic neural network (PNN) reaching 100%. The accuracy reported in previous works was measured in different datasets, making the comparison unfair.

### 4.2. Description of EEG Features 

This section describes the previously mentioned EEG feature details.

The cross-correlation (COR) corresponds to a measure of the similarity of two series as a function of the displacement of one relative to the other and the quadratic magnitude coherence (COH) between two variables, corresponding to the cross-spectral density function, which is derived from the FFT of cross-correlation normalized by their individual auto-spectral density functions. The imaginary part of the quadratic magnitude coherence (iCOH) is derived by keeping only the imaginary part of the complex numbers, which is the coherence [87].

The phase-locked value (PLV) characterizes the phase synchronization between two narrow-band signals, and the phase-locked index (PLI) is a measure of phase-lock that is zero in the case of linear mixing and nonzero when there is a consistent nonzero phase difference between the two signals. The p-index (RHO) quantifies the deviation of the cyclic relative phase distribution from the uniform distribution, approximating the probability density by the relative frequencies obtained with histograms of relative phases [87].

The transfer entropy (TE) measures the time-asymmetric transfer of information between two processes. Mutual information (MI) quantifies the amount of information that can be obtained about a random variable by observing another. Granger causality (GC) states that, for two simultaneously measured signals, one can predict the first signal better by incorporating the past information from the second signal than when only using information from the first signal. The directed transfer function (DTF) is similar to Granger causality but uses the elements of a different transfer matrix [87].

Complexity (Cx) consists of numerical information that is transformed into symbolic information after distinct words are created by decomposing symbolic sequences [91], which are encoded by the length of L(*n*). This feature can be defined by Equation (15):

(15) $$Cx=\frac{L\left(n\right)}{n}$$

The Higuchi fractal dimension (HFD) measures the self-similarity and irregularity of a time series. This feature is estimated using the slope of the linear fit over the log–log plot of the size and scales of the time series. The range of values is between 1 and 2. The Lyapunov exponents (Lya) show the average growing ratio of the primary distance between two neighboring points in the phase space [91]. Equation (16) can calculate this feature:

(16) $$\frac{\left\|\delta Xi\left(t\right)\right\|}{\left\|\delta Xi\left(0\right)\right\|}=2^{\lambda_{i}t}\left(t\to \infty \right)\;\lambda_{i}=\underset{t\to \infty }{\lim}\frac{1}{t}log_{2}\frac{\left\|\delta Xi\left(t\right)\right\|}{\left\|\delta Xi\left(0\right)\right\|}$$

where the distance between the point at time 0 is defined by $\left\|\delta Xi\left(0\right)\right\|$ and the point at time t is defined by $\left\|\delta Xi\left(t\right)\right\|$.

The phase space dynamics (PSD) of EEG signals can be transferred to successive triangles by averaging the coordinates of corners of each triangle ((*a_i_*, *a_i+_*_1_),(*a_i+1_*, *a_i+_*_2_),(*a_i+2_*, *a_i+_*_3_)), by which it is possible to obtain the centroid coordinate of the triangle. This coordinate is the same as that of Heron’s circle. The summation of distances between Heron’s circle (SDHC) [88] is computed as a graphical feature and is defined by Equation (17):

(17) $$\mathrm{PSDHC}=\overset{m-4}{\underset{i=1}{\sum }}\sqrt{{\left(\frac{a_{i+1}+a_{i+2}+a_{i+3}}{3}-\frac{a_{i}+a_{i+1}+a_{i+2}}{3}\right)}^{2}+{\left(\frac{a_{i+2}+a_{i+3}+a_{i+4}}{3}-\frac{a_{i+1}+a_{i+2}+a_{i+3}}{3}\right)}^{2}}$$

The summation of the shortest distance from each point relative to the 45-degree line (SH45) quantifies the data scatter rate around the 45-degree line. The SH45 measures the width of the PSD shape from the bisector of the first and third trigonometric regions, known as a line y = x [88]. It can be described by Equation (18):

(18) $$\mathrm{SH}45=\overset{m-1}{\underset{i=1}{\sum }}\frac{\left|a_{i+1}-a_{i}\right|}{\sqrt{2}}$$

The summation of the area of the triangles making successive points and the coordinate center (TACR) measures the variation rate of the PSD shape of the EEG signal [88]. The TACR is defined by Equation (19):

(19) $$\mathrm{TACR}=\overset{m-2}{\underset{i=1}{\sum }}\left|det\left[\begin{array}{ccc} 0 & a_{i} & a_{i+1}\\ 0 & a_{i+1} & a_{i+2}\\ 1 & 1 & 1 \end{array}\right]\right|$$

### 4.3. ERP Biomarkers in Schizophrenia

The EEG activity time-locked to stimuli is denominated event-related potentials (ERPs). ERPs are commonly used to capture neural activity related to sensory processes and consist of the averaged neural activity upon the repeated presentation of the same stimulus [92].

While studying the brain’s response to stimuli, participants might elicit spontaneous and involuntary neural activity during any moment of the recording. The neural response to stimuli is highly sensitive to the subject’s attention, the presence of motor acts, and inner thoughts, introducing random segments of activity in the signal that might even overshadow the targeted response. Conversely, the neural activity evoked by that particular stimulus will always be present at the moment of its presentation. Consequently, the spontaneous and variable activity will be filtered out by averaging activity across trials with the same stimulus, whereas the signal phase-locked to stimuli onset will become evident [83].

ERPs are widely used in EEG analysis since they are relatively simple to compute. Furthermore, classifiers are rarely used. To discriminate them, more straightforward statistical methods, such as Analysis of Variance (ANOVA), are often enough and constitute the majority of the methods used for this type of EEG analysis. As a result, the results presented here for using ERPs to diagnose schizophrenia are reported from the statistically significant differences found in both the latency and amplitude of these ERPs when comparing healthy subjects and patients to the diagnosis of schizophrenia within the mentioned studies.

**Figure 4:**
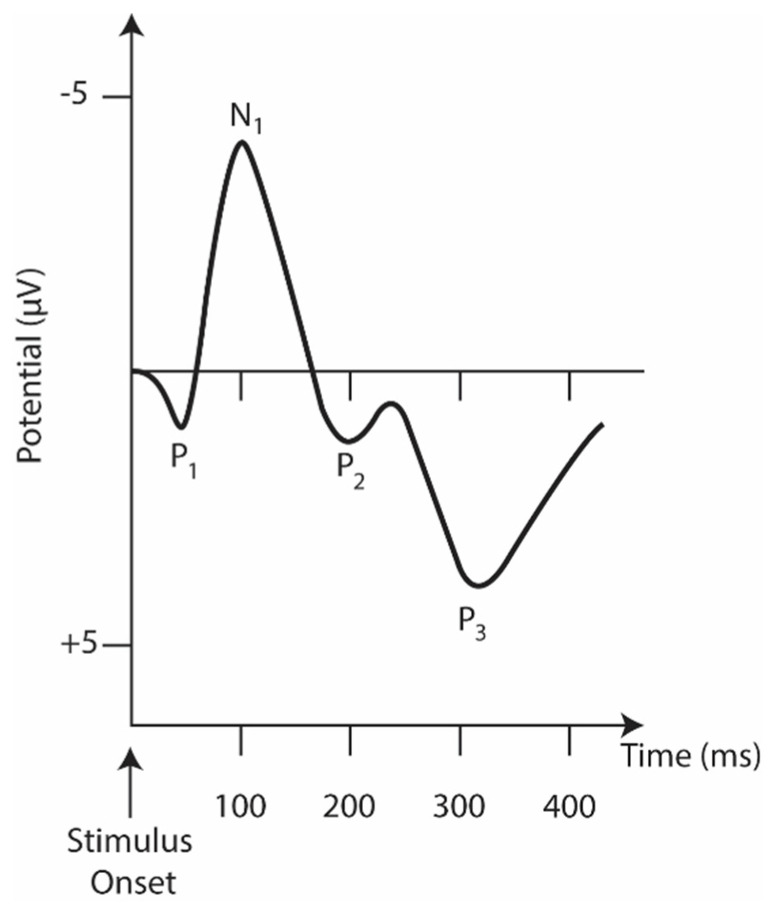
The idealized auditory ERP response is recorded at the head’s vertex. The *y*-axis is inverted where positive peaks are pointed downwards and negative peaks are pointed upwards.

In many cases, the auditory task (hearing a beep) is used to measure the cognitive decline in schizophrenia with ERP waveform alteration and reduced activity in specific cortical regions in schizophrenia [77]. Figure 4 represents the main ERP biomarkers.

Mismatch negativity (MMN) is a component of ERP or an event-related magnetic field (ERMF) that occurs in response to unexpected and rare stimuli in the surrounding environment. It is considered an important parameter for neuropsychiatric disorders and for schizophrenia, in particular [93].

The N1 component consists of a negative deflection at about 100 ms [76]. It is evident when an unexpected stimulus is presented [79]. A reduced N1 amplitude during vowel vocalization compared to passive listening and directed inner speech compared to a silent condition is seen in controls but not in patients [94,95].

Of the various ERPs (Figure 4), the components P50 (or Pa), N1, MMN, and P3 have received the most attention, as they are reliably impaired in schizophrenia and are, therefore, considered the most promising biomarker data [90].

The P50 (or Pa) is the earliest and smallest ERP component in auditory amplitude, reaching a general positive peak between 40 and 75 ms [96]. It is used to measure sensory switching using a conditioning test paradigm that involves the repeated presentation of a pair of auditory stimuli, S1 (condition) and S2 (test). The increased amplitude measurement (S2/S1) in patients is well established in the literature [97] and is related to their inability to filter the incoming flow of information and protect the brain from information overload [98]. Although its association with neuropsychological processes is still ambiguous [99], the P50 and S2 amplitude ratios have been linked to performance and attention [100]. In addition, P50 suppression impairment seems to be present in the risk phase, the prodromal phase, and the first episode [101].

The P3 component reflects information processing associated with attention and memory mechanisms [90]. For auditory stimuli, it consists of a positive peak deflection of 250–400 ms in adulthood [79]. Still, its latency and amplitude vary significantly depending on biological factors (e.g., genetics, intelligence, age, and smoking status, among others) [90]. A P3 component is triggered during oddball tasks in which multiple stimuli are presented and one of them occurs infrequently. A fair amount of research involves this ERP component as a P3 amplitude deficit, especially when evoked by auditory stimuli [78]. This is considered the most consistent and robust finding in schizophrenia [78,102,103].

Mismatch Negativity (MMN), typically generated 100 to 250 ms after stimulus onset, can be used as an objective index of sound discrimination accuracy and auditory sensory memory [90]. It is generated by the brain’s automatic response to any change in auditory stimulation that exceeds a specific threshold, roughly corresponding to the behavioral discrimination threshold [90]. Impoverished MMN production, reflected in attenuated amplitudes, is also a consistent finding in schizophrenia [104]. Interest is growing in studying MMN impairment with more complex paradigms (e.g., multiple sensory dimensions, complex sounds, and changes in stimulation patterns). These complex paradigms activate more complex brain regions as opposed to simpler deviations (e.g., pitch, duration, and intensity) that activate lower levels of the auditory system [105].

Although both ERPs share common mechanisms, MMN and P3 most likely portray different dysfunctions in schizophrenia.

Recent studies [105] indicate that MMN deficits generated during auditory tasks contribute to 18.7% of the variance in P3 deficits when both are examined. This proves that the high-level attention-dependent cognitive deficits central to schizophrenia do not originate from potentially preceding impairments at lower sensory, perceptual, or cognitive processing levels [78].

Some works used temporal, demographic, and time-frequency features of EEG. Zhang et al. [92] employed temporal features N1, N1TD, P2, P2TD (TD is time duration), and an EEG baseline as well as demographic (education and age) and temporal frequency features (power spectrum). These features were taken from an EEG-ERP with 9 electrodes: Fz, FCz, Cz, FC3, FC4, C3, C4, CP3, and CP4 (see Figure 3).

Kim et al. [84] used microstate and conventional EEG features extracted from five regions of interest (ROI): left anterior (Fp1, F7, and F3), right anterior (Fp2, F4, and F8), left posterior (T7, C3, P7, P3, and O1), right posterior (C4, T8, P4, P8, and O2), and central (Fz, Cz, and Pz) (see Figure 3). However, ERPs allow healthy and schizophrenic subjects’ discrimination based on P3, MMN, or N1 biomarkers and resting state signal complexity. Statistical measures or oscillatory power are also successful [106].

A bibliographic review revealed that nine authors used EEG to extract the gamma wave power spectrum (30–80 Hz), visual evocation potentials, alpha oscillations, power spectral density, functional connectivity, P3, N170, gamma event-related synchronization and correlation, intra-regional phase synchronization, event-related potential, and the average relative power in the Delta (1–4 Hz) and theta (4–8 Hz) frequency bands [107].

## 5. Discussion and Conclusions

As schizophrenia is a heterogeneous disease with multiple abnormal mechanisms and causes, it is essential not to consider a sample as an analysis of the whole. This may produce inconclusive results. To solve this, the sample can be divided based on symptoms, comorbidity, or patient-specific diagnosis [108,109,110].

The databases used in other studies are collected by the authors themselves with a limited number of samples. The acquisition of the signals (speech and EEG) is one challenging aspect of these works, which explains why no databases have been found. This work aims to identify speech and EEG characteristics that allow the identification of subjects with and without schizophrenia.

### 5.1. Speech Features

Speech in schizophrenia is considered a component of negative symptoms [25,57]; thus, analyzing proper speech features with a machine learning tool may be a promising method to identify schizophrenia. However, the biggest challenge is discriminating schizophrenia against other mental disorders, especially those with overlapping symptoms, such as depression and bipolar disorders [109].

Schizophrenic speech is less fluent, contains more (and longer) pauses, and has less pitch variability (fundamental frequency for each syllable).

In most studies using speech to identify schizophrenia, acoustic and time domain parameters are extracted from continuous speech. Using speech as a biomarker has the advantage of indirectly reflecting neural modulation via motor and acoustic variation, revealing emotion and thought via linguistic content. It is also difficult to hide speech-related symptoms. In addition, studies of speech can be generalized to different languages due to the consistency of the structure of vocal anatomy.

To collect voices from patients diagnosed with schizophrenia, speech stimulation techniques, clinical interviews, free speech tasks, descriptions of pictures or duties, or reading tasks are implemented.

According to the literature review presented in the previous sections, several speech parameters may be used as features. In Table 3, it is possible to see the features with more influence on identifying schizophrenia via speech. The prosodic characteristics represent speech aspects (beyond phonemes) and concern the auditory qualities of sound. The spectral characteristics are obtained by converting the time-based signal into the frequency domain, and temporal characteristics are calculated directly on the temporal waveform.

By analyzing speech, the emotional state, an essential feature of schizophrenia, can be determined. Emotion recognition via speech is performed using the following steps: audio input, preprocessing, feature extraction, classification, and output (emotion).

**Figure 5:**
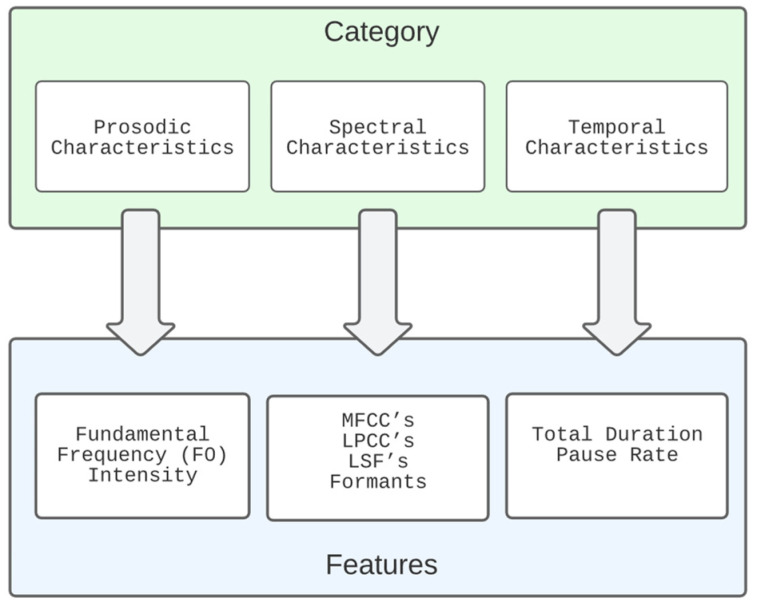
Features commonly used for detecting emotional state.

The emotional state is commonly evaluated by medical staff and is an essential feature of schizophrenia. Figure 5 presents the features most used for detecting emotional states. Although acoustic speech analysis is a promising method to identify schizophrenia situations, changing the approach to intelligent classifiers is challenging on many levels.

### 5.2. EEG Biomarkers

Currently, to identify schizophrenia, in addition to medical interviews (based on the PANSS scale or equivalent), EEGs, or more appropriately ERPs, are used.

The main disadvantage of using ERP is associated with its recording, in which patients are subjected to time-consuming EEG sessions involving multiple trials and possibly multiple paradigm conditions.

Most of the studied works used different electrodes. However, the most used electrodes were Fp2, F8, Fp1, F7, F4, Fz, O1, O2, C4, P4, P3, F3, C3, Cz, and Pz (Figure 3).

**Figure 6:**
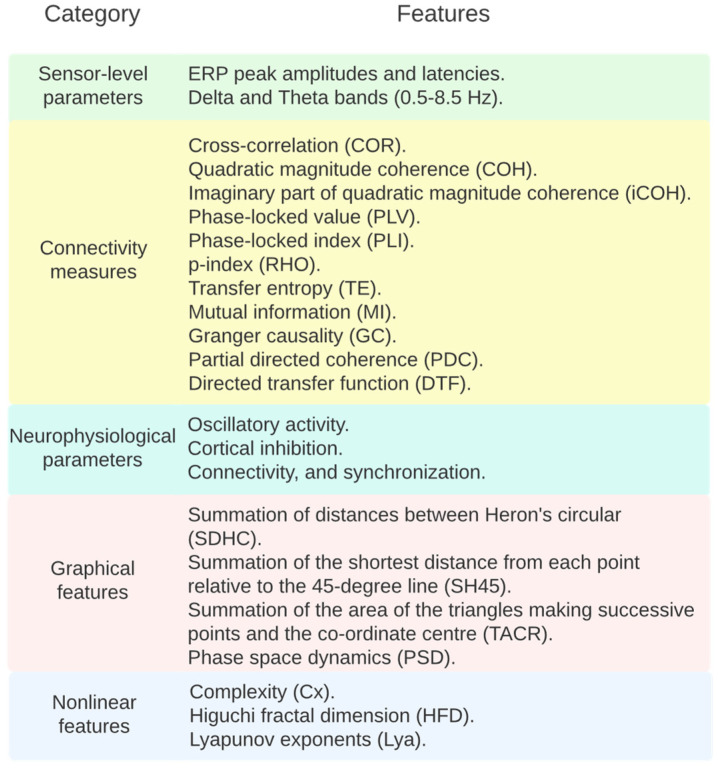
EEG features in schizophrenia.

The most promissory features of EEG are presented in Figure 6 and Table 4. The features are grouped into sensor-level parameters, connectivity measures, and neurophysiological, graphical, and non-linear features. They can vary between P3 ERP peak/latency, averaged cortical source activity [80], Partial Directed Coherence (PDC), the Direct Transfer Function (DTC) [87], complexity (Cx), the Higuchi fractal dimension (HFD), Lyapunov exponents (Lya) [91], the summation of distances between Heron’s circle (SDHC), the summation of the shortest distance from each point relative to the 45-degree line (SH45), and the summation of the area of the triangles making successive points and the coordinate center (TACR) [88]. Besides the [90] work that combines EEG with other techniques, the nonlinear features, such as Cx, HFD, and Lya, are the most promising ones since they achieved the highest accuracy (Table 4).

One of the most commonly used EEG parameters in the study of schizophrenia, P3 (Figure 4), is also altered in bipolar disorders [109] and depression [110].

**Figure 7:**
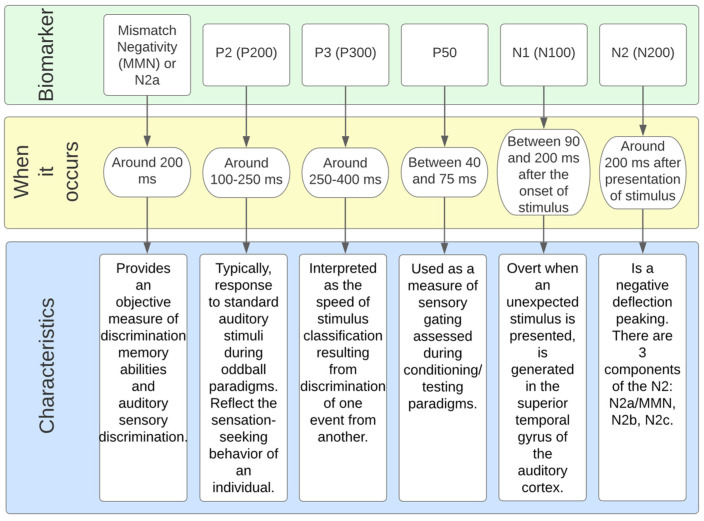
Biomarkers of ERP for schizophrenia detection.

Figure 7 illustrates the ERP biomarkers. According to our study, the ERP components presented in Figure 7 can be robust biomarkers to detect schizophrenia. In patients with schizophrenia, the MMN has a decrease in amplitude and abnormal topographical distribution. The P2, N1, and N2 components have a reduced amplitude. In people with schizophrenia, the P50 shows reduced suppression. One of the most robust ERP components in schizophrenia is P3, in which its amplitude decreases. The P3 amplitude is sensitive to alterations in positive symptoms. In the oldest patients, the P3 is generally smaller in amplitude and longer in latency.

### 5.3. Concluding Remarks

During this research, it was found that speech and EEG parameters can be promising for diagnosing schizophrenia. However, nowadays, the medical community accepts EEG parameters more often. What may condition both methods is the acquisition procedure. Although it is easier to conduct speech acquisition, the equipment used may not be of sufficient quality, and the environment may not have the right conditions for sound recording. Though obtaining an EEG is not an invasive procedure, placing something on the scalp of someone diagnosed with schizophrenia may cause the subject to exhibit unwanted behavior. Another disadvantage is the access to ECG equipment, particularly for research purposes.

The speech parameters can be grouped into three levels: speech acoustic features, semantic features, and emotional features. The most common acoustic features are divided into four main categories: the prosodic category (F0, intensity/loudness, amplitude, jitter, and shimmer), spectral category (F1, F2, F3 and MFCCs), temporal characteristics (utterance duration, duration of pauses, and number of pauses) and statistical measures (number of words and verbosity).

Concerning the accuracy achieved in previous works using speech features, the combination of at least two categories achieved higher scores in the accuracy in classifying schizophrenia subjects. The prosodic features were used in the works with higher accuracy when combined with spectral or temporal features. The work with higher accuracy used the prosodic and spectral features QEVA, SDVV, and SSDL that were derived from the F0 and spectrogram.

The most used EEG biomarkers are MMN, P2, P3, P50, N1, and N2, which are obtained from ERPs. The EEG features with higher accuracy in schizophrenia classification subjects are the nonlinear features, such as Cx, HFD, and Lya.

SVM is the machine learning tool used so far with the best research results.

Some researchers have also used other levels of parameters, such as text features and body movements.

This research contributed to identifying the best set of features to use in future developments in the direction of the identification of schizophrenia with machine learning tools as a support system.

## Figures and Tables

**Table 1:** Speech features used to identify schizophrenia.

Category of Feature	Feature	Work
Prosodic Characteristics	F0/Pitch	[4,12,17,26,28,34,42,47,55,56]
	Intensity/Loudness/Amplitude	[4,12,17,25,26,28,45,47,56]
	Jitter Shimmer	[12,24,45]
	HNR	[42]
	NHR	[45]
	Quantization Error and Vector Angle (QEVA); Standard Dynamic Volume Value (SDVV)	[48]
	Articulation rate	[39,52]
	Peak slope	[2]
Spectral Characteristics	MFCCs	[4,12,17,28,45]
	F1 F2	[4,12,17,26,34,45,56]
	F3	[4,12,17,25,45,56]
	Line Spectral Frequencies (LSF);	[55]
	Linear Predictive Coefficients (LPC)	[2]
	Symmetric Spectral Difference Level (SSDL)	[48]
Temporal Characteristics	Zero-crossing rate	[24,27]
	Duration of pauses	[2,12,42,43,47,49,57]
	Utterance duration	[4,17,24,40,43,44,47,49,58]
	Number of pauses	[43,45,47]
	Gap duration	[25,43]
	The proportion of silence	[12,49]
	Total recording time	[47]
	Voiced/unvoiced percentages; voiced/unvoiced ratio; velocity of Speech	[45]
Statistical Measures	Quasi-open Quotient (QOQ)	[2]
	Number of words; verbosity (use of excessive words)	[31,44,57]
	Speaking turns, interruptions, and interjections	[12,46]
	Probability of voicing	[55]
	IQR (interquartile range) of MFCCs and F0 variation	[45]
	Skewness and kurtosis (of log Mel freq. band); mean value (of waveform Correlation, jitter, and shimmer), slope sign changes	[24,57]
	Third, fourth, and fifth moments; Hjorth parameter activity; mobility and complexity; waveform length	[56]
	Minimum semantic distance for first-order coherence; mean semantic distance for first-order coherence	[50]
	Pitch range; standard deviation of pitch; power standard deviation; mean waveform correlation	[24]

**Table 2:** Accuracy of the speech features in the classification.

Number of Used Categories	Categories	Ref.	Accuracy (%)
1	Prosodic	[39]	To evaluate the relative contributions of motor and cognitive symptoms on speech output in persons with schizophrenia
	Temporal	[27]	Language and thought disorder in multilingual schizophrenia
		[40]	Understanding constricted affect in schizotypal via computerized prosodic analysis,
		[43]	80
		[49]	They identified weak untypicalities in pitch variability related to flat affect and stronger untypicalities in proportion of spoken time, speech rate, and pauses related to alogia and flat affect.
		[58]	93.8% (emotion detection)
	Statistical	[31]	They characterized the relationship between structural and semantic features, which explained 54% of negative symptoms variance.
		[46]	93
		[50]	100 (psychotic outbreaks in young people at CHR).
		[57]	87.56
2	Prosodic and Spectral	[26]	The authors used such methods to understand the underpinnings of aprosody.
		[28]	79.49
		[34]	F2 was statistically significantly correlated with the severity of negative symptoms.
		[48]	98.2
	Temporal and Statistical	[44]	85
	Prosodic and Temporal	[42]	93.8
		[47]	79.4
	Acoustic and Text Features	[52,53]	76.2
3	Prosodic, Spectral, and Temporal	[4]	81.3
		[17]	90.5
		[25]	91.79
	Prosodic, Spectral, and Statistical	[55]	82
		[56]	The association between disorganized speech and adjunctive use of mood stabilizers could perhaps be understood in the context of a relationship with impulsiveness/aggressiveness or in terms of deconstructing the Kraepelinian dualism.
	Prosodic, Temporal, and Statistical	[24]	87.5
4	Prosodic, Spectral, Temporal, and Statistical	[2]	The authors provide an online database with their search results and synthesize how acoustic features appear in each disorder.
		[12]	90
		[45]	90

**Table 3:** Features with more influence on the identification of schizophrenia via speech.

Prosodic Characteristics	Fundamental frequency (F0) or Pitch Intensity/loudness
Spectral Characteristics	First, second, and third order formants (F1, F2, and F3) Mel-Frequency Cepstral Coefficients (MFCCs)
Temporal Characteristics	Duration of pauses and sentences. Duration of the interval between words. * Quantitatively, the quantity of words/pauses can be considered (it is essential to consider that this quantity can derive from several factors, such as the stimulation task used or the individual’s schooling).

**Table 4:** References and accuracy obtained with EEG features.

Ref.	Accuracy (%)	Features
[85]	100	Combination of FMRI-SMRI-EEG
[80]	78.24	Combined sensor-level and source-level EEG features
[87]	82.36	Connectivity measures
[91]	100	Nonlinear features: complexity (Cx), Higuchi fractal dimension (HFD), and Lyapunov exponents (Lya)
[88]	94.8	Phase space dynamic (PSD)
[8]	99.47	Collatz pattern technique
[92]	74.07	Features extracted from event-related potential (ERP).
[84]	75.64	Microstate features (duration, occurrence, and coverage), conventional EEG features (statistical, frequency, and temporal characteristics)

## Data Availability

Not applicable.

## Abbreviations

**Abbreviation**	**Meaning**
AI	artificial intelligence
AVHs	auditory verbal hallucinations
BPRS	Brief Psychiatric Rating Scale
CD	correlation dimension
CHR	high clinical risk
CNN	convolutional neural network
COH	quadratic magnitude coherence
COR	cross-correlation
Cx	complexity
DTF	directed transfer function
EC	effective connectivity
EEG	electroencephalography
ERP	event-related potentials
F0	fundamental frequency
F1, F2, F3	frequency formants
FC	functional connectivity
fMRI	functional magnetic resonance imaging
GC	Granger causality
GNE	glottal noise excitation
GRNN	generalized regression neural network
HE	Hurst exponent
HFD	Higuchi fractal dimension
HNGD	Hilbert envelope of numerator group delay
HNR	Harmonic-to-noise ratio
I	intensity/loudness
iCOH	imaginary part of quadratic magnitude coherence
INCA	iterative neighborhood component analysis
IQR	interquartile range
KNN	k nearest neighbors
LDA	linear discriminant analysis
LIWC	Linguistic Inquiry and Word Count
LLD	low-level descriptors
LLE	largest Lyapunov exponent
LPCC	linear prediction cepstral coefficients
LSA	latent semantic analysis
LSF	linear spectral features
LSTM	long-short-term memory network
Lya	Lyapunov exponents
MCCA	multi-set canonical correlation analysis
MFCC	Mel frequency cepstral coefficients
MI	mutual information
ML	machine learning
MLP	multi-layer Perceptron
MMN	mismatch negativity
NHR	noise to harmonic ratio
NNE	normalized noise energy
NSA	Negative Symptom Assessment
PANSS	Positive and Negative Syndrome Scale
PDC	partial directed coherence
PLI	phase-locked index
PLP coefficients	perceptual linear lrediction
PLV	phase-locked value
PSD	phase space dynamics
PSO	particle swarm optimization
QEVA	quantification error and vector angle
QOQ	quasi-open quotient
RHO	p-index
ROI	regions of interest
SANS	Scale for the Assessment of Negative Symptoms
SC	structural connectivity
SD	duration of pauses and sentences
SDHC	summation of distances between Heron’s circular
SDVV	standard dynamic volume value
SH45	summation of the shortest distance from each point relative to the 45-degree line
sMRI	structural magnetic resonance imaging
SSDL	symmetric spectral difference level
SVM	support vector machine
TACR	summation of the area of the triangles making successive points and the coordinate centre
TE	transfer entropy
WoS	Web of Science
ZTW	zero time windowing
